# Midfacial degloving - acess to nasal cavity and paranasal sinuses lesions

**DOI:** 10.1016/S1808-8694(15)30050-1

**Published:** 2015-10-19

**Authors:** Lidiane Maria de Brito Macedo Ferreira, Adson Sales do Nascimento Rios, Érika Ferreira Gomes, Jorge Ferreira Azevedo, Roberta de Paula Araújo, Robiany Barbosa Moraes

**Affiliations:** a ENT resident - Hospital Geral de Fortaleza-SESA/SUS.; b ENT resident - Hospital Geral de Fortaleza-SESA/SUS.; c Otolaryngologist, Preceptor of Otolaryngology - Hospital Geral de Fortaleza-SESA/SUS.; d Oncologist surgeon, Head of the head and neck department - Hospital Geral de Fortaleza-SESA/SUS.; e ENT resident - Hospital Geral de Fortaleza-SESA/SUS.; f Registered Nurse graduated from UNIFOR/CE.

**Keywords:** Midfacial degloving

## Abstract

Common surgical approaches for medial maxillectomy include lateral rhinotomy and midfacial degloving. Lateral rhinotomy provides excellent surgical exposure but leaves a bulging scar on the face. Despite its own limitations, midfacial degloving has been preferred to lateral rhinotomy because it does not leave any external scar on the face^1^. The aim of this study is to evaluate the cosmetic results and surgical exposure access of midfacial degloving. Treatment morbidity was evaluated through: post operative hospital stay length, blood transfusion needs, complications, pre and post operative hemoglobin levels, disease recurrence, nasal packing, type of suture and antibiotics. Retrospective study was carried out with sixteen patients treated at the Hospital Geral de Fortaleza SESA/SUS from December 1999 through November 2003. Based on the results, we may conclude that midfacial degloving is effective to treat extensive nasal cavity lesions and paranasal sinuses with reduced post operative morbidity.

## INTRODUCTION

Nasosinusal tumors, specially those with important invasive components, require an aggressive and broad surgical management, one that allows the surgeon to have a clear view of the tumor margins so that the procedure may become as curative as possible. Conventional maxillectomy, lateral rhinotomy and the Weber-Fergusson or Diffenbach approaches are still worldly used; however in many cases they have been replaced by the mid-facial degloving, which avoids facial scars. This approach has been used for about 25 years now and its use is increasing in the treatment of extensive benign lesions of the rhinosinusal region, for some malignant neoplasms of this area, and also to provide access to the nasopharynx and infratemporal fossa^2^.

The surgical approach is the following:

After orotracheal intubation, nasal topic vasoconstrictor drops and local infiltration, the procedure starts with a transfixating incision and a bilateral intercartilaginous incision. Nasal dorsum tissues, anterior wall of the maxillary sinus, glabella and frontal bone are lifted through the intercartilaginous incision, they are then extended laterally towards the nasal cavity floor until they touch the caudal portion of the transfixating incision bilaterally, finishing the incision in a circle. Afterwards, a sublabial incision is carried out from the first molar, all the way to the corresponding contralateral tooth. This incision hits the muco-periosteum and continues with the intranasal incision in the nostril region. A periosteum elevator is used to raise the tissues bilaterally until one reaches the inferior orbital rim, taking the necessary care to protect the infraorbitary nerve and vessels. The flap, which includes the inferior lateral cartilage and the columellae, is raised all the way to the glabella, medial cantus region and forehead, in a way as to expose all the mid-facial skeleton^3^.

For what we showed by the technique description, the degloving approach bears the great advantage of exposing all the intranasal and nasosinusal structures for surgical intervention, and this represents a decisive factor when one considers malignant diseases, in which the margins have to be free, and besides, it bears excellent cosmetic results because there is no skin incision.

## OBJECTIVE

Our goal is to describe the cosmetic results and those of a better intraoperative access for nasosinusal surgeries via degloving, as well as assessing the post-operative morbidity of patients who underwent this procedure, we carried out a study with the patients admitted to the Hospital Geral de Fortaleza SESA/SUS, bearing nasoangiofibroma, nasosinusal inverted papilloma, stesioneuroblastoma, cystic adenoid carcinoma, clivus chordoma, cholesterol granuloma and gigantic cells granuloma.

## MATERIALS AND METHODS

In order to assess post-operative morbidity and cosmetic results based on the surgical access of patients operated through degloving, we carried out a retrospective study of 16 patients operated at the Hospital Geral de Fortaleza SESA/SUS, from December 1999 to November of 2003, at the Head and Neck Department, under general anesthesia. The patients had nasoangiofibroma (9 patients), nasosinusal inverted papilloma (2 patients), stesioneuroblastoma (1 patient), cystic adenoid carcinoma (1 patient), clivus chordoma (1 patient), cholesterol granuloma (1 patient) and giant cell granuloma (1 patient), confirmed by histopathology. This research was carried out with medical chart assessment from the hospital archives and the filling out of a standardized protocol including the following data: name, chart number, date of surgery, lesion topography, pre-operative hemoglobin, post-operative hospital stay, use of antibiotic agents, nasal packing duration, surgical wire used, need for blood transfusion, complications and follow up time without recurrence (through nasofibroscopy).

## RESULTS

The patients in this series had average age of 25.5 years (varying between 12 and 76 years) (Table 1). All the patients required nasal packing with average duration of 4.5 days (between 2 and 9). In 15 patients there was a reduction on the hemoglobin levels at an average of 2.07mg/dL (between 0.7 and 4.3 mg/dL). All angiofibroma patients presented reductions in blood count levels, at an average of 2.01mg/dL (0.7 - 3.8mg/dL). Nine patients required blood transfusion during surgery, of which 6 had angiofibroma, and the average volume of blood transfused was of 700mL (between 300-1,200mL). All patients used prophylactic or therapeutic antibiotics, and the average use was of 6.5 days (between 3–12 days). Of the antibiotics used, the most common was Cephalothin (10 of the 16 patients) (Table 2). In order to suture the nasal vestibule and the oral mucosa, we used Vicryl® 4.0 wire in 15 patients. Only 1 patient was sutured with Monocryl 4.0 suture wire, and in two patients we used both Vicryl 4.0 and Silk 4.0. There were complications in 7 patients, all minor. So far, only 2 of the 16 patients had recurrence of the disease they were operated for. The average hospital stay was of 7.3 days (between 4–16 days) post-op (Table 3). The post-op follow up was carried out with flexible video-nasofibroscopy, and all diagnoses were confirmed by histopathology.

**Table 1:** Relation of patients, surgery performed, age and lesion topography.

Patient	Surgery	Age	Lesion topography
1	Resection of nasopharyngeal angiofibroma	14 years old	Nasopharynx
2	Resection of nasopharyngeal angiofibroma	19 years old	Nasopharynx
3	Medial ethmoidectomy and maxillectomy	15 years old	Nasal cavity
4	Resection of nasopharyngeal angiofibroma	18 years old	Nasopharynx
5	Resection of clivus chordoma	31 years old	Clivus
6	ethmoidectomy and maxillectomy	57 years old	Maxillary sinus
7	Resection of nasopharyngeal angiofibroma	20 years old	Nasopharynx
8	Resection of nasopharyngeal angiofibroma	20 years old	Nasopharynx
9	Resection of nasopharyngeal angiofibroma	16 years old	Nasopharynx
10	Resection of nasopharyngeal angiofibroma	15 years old	Nasopharynx
11	Resection of nasopharyngeal angiofibroma	19 years old	Nasopharynx
12	Maxillectomy	26 years old	Maxillary sinus
13	Resection of nasosinusal inverted papilloma	76 years old	Nasal cavity
14	Resection of nasosinusal inverted papilloma	42 years old	Nasal cavity
15	Resection of stesioneuroblastoma	12 years old	Nasal cavity
16	Resection of nasopharyngeal angiofibroma	23 years old	Nasopharynx

**Table 2:** Relation of pre and post operative hemoglobin, blood transfusion, nasal packing and antibiotics.

Patient	Hb pre-op	Hb post-op	Transfusion	Nasal packing	Antibiotic
1	12,0	10,9	900 ml	3 days	Cephalothin 7 days
2	12,1	11,4	No	2 days	Cephalothin 7 days
3	11,5	12,8	600 ml	2 days	Cephalothin 5 days
4	15,5	12,0	600mL	6 days	Cephalothin 7 days
5	13,2	8,93	1200ml	5 days	Cephtriaxon 10 days
6	14,5	11,4	No	3 days	Ampicilin 12 days
7	12,7	11,0	600ml	3 days	Cephalothin 06 days
8	16,0	14,7	300ml	3 days	Cephalothin 03 days
9	16,3	12,5	300mL	5 days	Cephalothin 3 days
10	15,2	13,8	No	6 days	Cephalexin 10 days
11	12,0	10,0	No	4 days	Cephalothin 04 days
12	13,9	12,7	No	9 days	Cephalexin 10 days
13	14,0	12,7	No	5 days	Clindamicina 04 days
14	12,4	10,6	600mL	4 days	Clindamycin/Amycacin 06 days
15	14,0	12,5	No	5 days	Cephalothin 07days
16	11,6	9,0	1200ml	3 days	Cephalothin 03 days

**Table 3:** Relation of the surgical wire used in oral and nasal mucosas, hospital stay after surgery, recurrence and complications.

Patient	Surgical wire	Hospital stay	Recurrence	Complications
1	Vicryl 4.0	7 days	No. 55 months	Bleeding
2	Vicryl 4.0	7 days	No. 40 months	No
3	Vicryl 4.0	5 days	Yes. 29 months	No
4	Vicryl 4.0	7 days	No. 31 months	Suture dehiscence
5	Vicryl 4.0	16 days	Yes. 6 months	No
6	Vicryl 4.0	12 days	No. 37 months	Oroantral fistula
7	Vicryl 4.0	6 days	No. 28 months	Nasal alae drop
8	Vicryl 4.0 and Silk 4.0	6 days	No. 24 months	Trismus
9	Vicryl 4.0 and Silk 4.0	6 days	No. 24 months	No
10	Vicryl 4.0	7 days	No. 21 months	Epistaxis and septum perforation
11	Vicryl 4.0	4 days	No. 19 months	No
12	Vicryl 4.0	10 days	No. 9 months	Trismus
13	Monocryl 4.0	5 days	No. 15 months	No
14	Vicryl 4.0	6 days	No. 12 months	No
15	Vicryl 4.0	8 days	Yes. 6 months	No
16	Vicryl 4.0	5 days	No. 8 months	No

## DISCUSSION

Lateral rhinotomy is a traditional approach for nasal cavity and paranasal sinus tumor surgeries. This approach provides an excellent surgical exposure; notwithstanding, even with such advantage, its use is limited, because it leaves a prominent scar on the face. The degloving approach was first described in 1974, by Casson et al.^1^ and has become very popular because of its major advantages of avoiding facial incisions and providing bilateral exposure of the nasal cavity. Thus, the mid-facial degloving approach has been used as a first option for medial maxillectomy, radical maxillectomy and non-complicated cranio-facial surgeries^4^, ^5^, ^6^.

**Figure 1:**
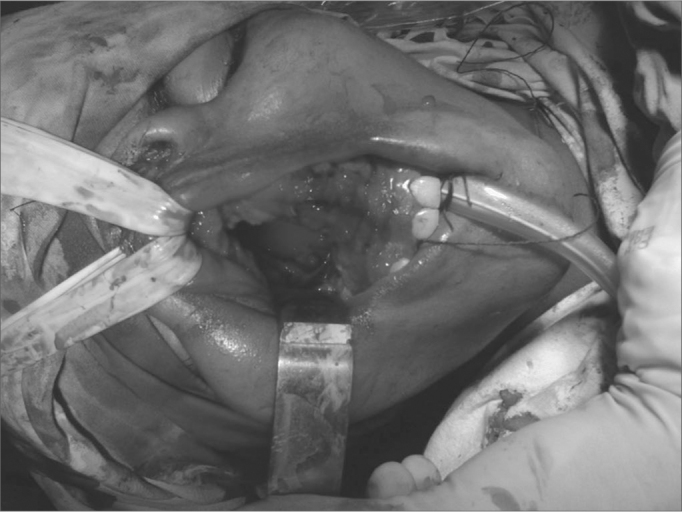
Approach to nasoangiofibroma through mid-facial degloving.

**Figure 2:**
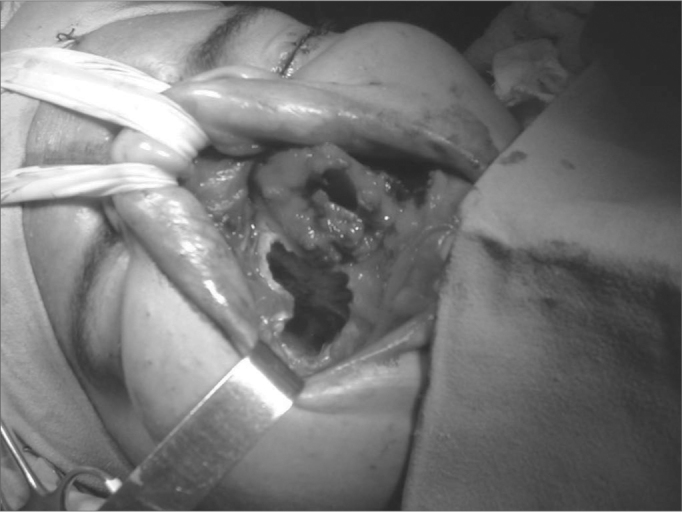
Approach to stesioneuroblastoma through mid-facial degloving.

Some changes to the degloving approach have been described, in order to avoid vestibular stenosis, which is the most frequent and significant complication^7^.

The standard procedure comprises an extensive gengivobuccal incision, a transfixating septal incision, an intercartilaginous incision and an incision in the nostril. The vestibular stenosis occurs as a consequence of the circumferential incision that is made in the nasal vestibule during the procedure.

Of the sixteen patients operated in our department, there were post-operative complications in seven, but none of them related to nasal vestibule stenosis, thus showing that the surgical procedure in itself, based on surgeon’s experience and the wire used (Vicryl® in most of the cases) provides good cosmetic outcome. Only one patient had suture dehiscence. The suture wire used in the vestibular region is an important analysis point to be considered, in order to guarantee the success of the procedure as far as physiology and nasal cosmetics are concerned^8^.

Although it has been already established that one hour more of surgery time increases by twofold the incidence of infection and it is certainly one more factor related to an increase in injuries and their repercussion, operative time is still debated. There is no relationship between surgery time and post-operative complications, death or long term survival.

Thus, it is known that one of the inconveniencies of the type of surgery under study is that it takes longer than its endoscopic counterpart, and the latter is a feasible and very much efficient alternative for the treatment of nasal cavity tumors in their initial stage, because it is less aggressive and brings less complications to the patient’s post-op recovery. Notwithstanding, considering larger lesions, even if benign, the endoscopic approach is not adequate.

Another drawback is that since this represents an extensive surgery, there is more bleeding, requiring a greater need for volume replacement, thus 9 of the 16 patients received red blood cells concentrate transfusion, at an average of 700mL/patient, and there was a fall in hemoglobin levels in 15 patients, at an average of 2.07mg/dL. However, it is worth highlighting that of these nine, six patients had angiofibroma (the average blood volume transfused was 650mL of red blood cells concentrate), a vascular lesion that by itself causes active bleeding, and from the nine patients with angiofibroma, all had reductions in their blood element counts. All patients with this lesion underwent tumor embolization before surgery. In assessing bleeding of the remaining tumors, 4 patients did not require blood transfusion and 3 required an average of 800mL. Therefore, the assessment of intra-operative bleeding is much more related to tumor histology type then the surgical technique used. Although the literature shows that intraoperative blood transfusion may be an indicator of bad prognosis - reducing post-operative survival, this has not been found in the present study.

Besides, nasal packing was necessary in all patients, with an average of 4.5 days in hospital stay. Even then, the post-operative hospitalization time was of 7.3 days in average, and only 2 patients had recurrent disease. These two factors alone, coupled to the advantage of not leaving scars, shows that although degloving does have the aforementioned inconvenient points, it is efficient in the treatment of extensive lesions involving the nasal cavity and paranasal sinuses.

## CONCLUSION

With the data presented in this study, we may conclude that the degloving approach to resect nasosinusal tumors is effective and bears the advantages of broad surgical exposure, excellent cosmetic results, very low post-operative complication rates and low recurrence rates.
